# Solution of nonlinear higher-index Hessenberg DAEs by Adomian polynomials and differential transform method

**DOI:** 10.1186/s40064-015-1443-3

**Published:** 2015-10-29

**Authors:** Brahim Benhammouda

**Affiliations:** Higher Colleges of Technology, Abu Dhabi Men’s College, P.O. Box 25035, Abu Dhabi, United Arab Emirates

**Keywords:** Differential-algebraic equations, Adomian polynomials, Differential transform method, Padé approximants, Hessenberg DAEs

## Abstract

The solution of higher-index Hessenberg differential-algebraic equations (DAEs) is of great importance since this type of DAEs often arises in applications. Higher-index DAEs are known to be numerically and analytically difficult to solve. In this paper, we present a new analytical method for the solution of two classes of higher-index Hessenberg DAEs. The method is based on Adomian polynomials and the differential transform method (DTM). First, the DTM is applied to the DAE where the differential transforms of nonlinear terms are calculated using Adomian polynomials. Then, based on the index condition, the resulting recursion system is transformed into a nonsingular linear algebraic system. This system is then solved to obtain the coefficients of the power series solution. The main advantage of the proposed technique is that it does not require an index reduction nor a linearization. Two test problems are solved to demonstrate the effectiveness of the method. In addition, to extend the domain of convergence of the approximate series solution, we propose a post-treatment with Laplace-Padé resummation method.

## Background

Differential-algebraic equations (DAEs) are used to describe many physical problems. These types of equations arise for instance in the modelling of electrical networks, optimal control, mechanical systems, incompressible fluids and chemical process simulations. An important quantity that characterizes DAEs and which plays a key role in the treatment of these equations is the index. There are various definitions for the index of a DAE (Martinson and Barton 2000; Günther and Wagner 2001; Rang and Angermann 2005; Kunkel and Mehrmann 1996) but the most used one is the differentiation index. It is defined as the minimum number of times that all or part of the DAE must be differentiated with respect to time, in order to obtain an ordinary differential equation (Martinson and Barton 2000). Higher-index DAEs (differentiation index greater than one) arise naturally in many important application problems. For instance, they model constrained multibody systems (Simeon 1993, 1996; Benhammouda and Vazquez-Leal 2015), vehicle system dynamics (Simeon et al. 1991, 1994), space shuttle simulation (Brenan 1983) and incompressible fluids. Unfortunately, these DAEs are known to be difficult to solve, even with numerical methods, due to their complex structure. One reason for this; solutions of higher-index DAEs are constrained for all time by some hidden algebraic equations. As a consequence, initial conditions cannot be prescribed arbitrarily for all solution components as they have to fulfill the constraint equations. Therefore, to start the numerical integration, we need to compute some consistent initial conditions. That is to determine those initial conditions which satisfy all the constraints in the system. Using inconsistent initial conditions or poor estimates can cause the solution of the DAE to drift off the constraints manifold and lead to a non physical solution. Since numerical integration methods have difficulties in solving higher-index DAEs, these problems are usually dealt with by first transforming them to ordinary differential systems (index-zero) or index-one DAEs before applying numerical integration methods. This procedure, known as index-reduction, can be very expensive and may change the properties of the solution of the original problem. Therefore, since important application problems in science and engineering often lead to higher-index DAEs, new techniques are needed to solve these DAEs efficiently.

Over the past decades, significant progress has occurred in the solution of DAEs. Some of these works have focused on the numerical solution and include backward differentiation formula (Brenan 1983), Runge Kutta method (Hairer et al. 1989), pseudospectral method (Hosseini 2005) and finite differences method (Wu and White 2004). One can find other methods for the solution of DAEs like blended implicit methods (Brugnano et al. 2006), implicit Euler (Sand 2002), Chebyshev polynomials (Husein and Jaradat 2008), and arbitrary order Krylov deferred correction methods (Huang et al. 2007).

In recent years, some analytical approximation methods have been developed to solve DAEs. Among such techniques one can find the Adomian decomposition method (ADM) (Hosseini 2006; Celik et al. 2006), the homotopy perturbation method (HPM) (Soltanian et al. 2010; Salehi et al. 2012), the variational iteration method (VIM) (Karta and Celik 2012), the homotopy analysis method (HAM) (Awawdeh et al. 2009), the Padé method (Celik and Bayram 2003) and the differential transform method (DTM) (Benhammouda and Vazquez-Leal 2015; Liu and Song 2007; Ayaz 2004). The ADM, Adomian polynomials and DTM were also applied to solve many other problems. The ADM, for example, was used in computing solutions of algebraic equations (Adomian and Rach 1985; Fatoorehchi et al. 2014a, b, 2015; Fatoorehchi and Abolghasemi 2014a, b; Fatoorehchi et al. 2015b, d, c). The ADM and Adomian polynomials were applied to various problems in engineering fields (Fatoorehchi et al. 2015f, g, c; Fatoorehchi and Abolghasemi 2015, 2013b). Recently, the DTM was used as a new tool to compute Laplace transforms to solve many problems (Fatoorehchi et al. 2015a; Fatoorehchi and Abolghasemi 2012).

In this work, we present a new procedure for solving nonlinear higher-index Hessenberg DAEs. The method is based on Adomian polynomials (Rach 1984, 2008; Wazwaz 2000; Duan 2010a, b, 2011) and the DTM (Odibat et al. 2010; Lal and Ahlawat 2015; El-Zahar 2013; Fatoorehchi and Abolghasemi 2013a; Gökdoğan et al. 2012; Benhammouda et al. 2014). The DTM is first applied to the DAE where the differential transforms of nonlinear terms are found using Adomian polynomials to obtain a recursion system for the power series coefficients. Based on the index condition, a nonsingular linear recursion system is then derived and solved. It is important to note that the developed procedure does not require an index-reduction nor a linearization. Also it does not depend on complicated tools like perturbation parameters, trial functions, or Lagrangian multipliers as required for perturbation method, HPM or VIM. To enlarge the domain of convergence of the truncated power series, we apply a post-treatment based on Laplace-Padé resummation method (Benhammouda et al. 2014; Torabi and Yaghoobi 2011; Raftari and Yildirim 2011; Bararnia et al. 2012; George A Baker et al. 1996; Vazquez-Leal et al. 2012; Vazquez-Leal and Guerrero 2014; Khan et al. 2013; Benhammouda et al. 2014).

Two examples of nonlinear higher-index Hessenberg DAEs are solved to demonstrate the effectiveness of the proposed method. Finally, our procedure is straightforward and can be programmed in Maple or Mathematica.

This paper is organized as follows: in "Differential transform method", we review the DTM. Next, in "Padé approximant", "Laplace-Padé resummation method" and "Adomian polynomials and their relation with DTM" we give the basic concepts of Padé approximants, Laplace-Pad é resummation method and Adomian polynomials and their relation with DTM. In "Solution of higher-index Hessenberg DAEs by Adomian polynomials and DTM", we present our analytical method for the solution of nonlinear higher-index Hessenberg DAEs. Then in "Cases study", we apply the developed method to solve two nonlinear higher-index Hessenberg DAEs. Finally, a discussion and a conclusion are given in "Discussion" and "Conclusion", respectively.

## Differential transform method

For convenience of the reader, we will review the DTM (Odibat et al. 2010; Lal and Ahlawat 2015; El-Zahar 2013; Fatoorehchi and Abolghasemi 2013a; Gökdoğan et al. 2012) and show how this method is used to solve ordinary differential equations.

### **Definition 2.1**

If a function *u*(*t*) is analytical with respect to *t* in the domain of interest, then

1 $$\begin{aligned} U_{k}=\frac{1}{k!}\left[ \frac{d^{k}u(t)}{dt^{k}}\right] _{t=t_{0}}, \end{aligned}$$

is the transformed function of *u*(*t*).

### **Definition 2.2**

The differential inverse transforms of the set $\left\{ U_{k}\right\} _{k=0}^{n}$ is defined by

2 $$\begin{aligned} u(t)=\displaystyle {{\sum _{k=0}^{\infty }}}U_{k}\left( t-t_{0}\right) ^{k}. \end{aligned}$$

Substituting (1) into (2), we deduce that

3 $$\begin{aligned} u(t)=\displaystyle {{\sum _{k=0}^{\infty }}}\frac{1}{k!}\left[ \frac{d^{k}u(t) }{dt^{k}}\right] _{t=t_{0}}\left( t-t_{0}\right) ^{k}. \end{aligned}$$

From the above definitions, it is easy to see that the concept of the DTM is obtained from the power series expansion. To illustrate the application of the DTM to solve ordinary differential equations, we consider the nonlinear equation

4 $$\begin{aligned} \frac{du(t)}{dt}=f\left( u(t),t\right) , \quad t\ge t_{0}, \end{aligned}$$

where $f\left( u(t),t\right)$ is a nonlinear smooth function.

Equation (4) is supplied with some initial condition

5 $$\begin{aligned} u(t_{0})=u_{0}. \end{aligned}$$

DTM establishes that the solution of (4) can be written as

6 $$\begin{aligned} u(t)=\displaystyle {{\sum _{k=0}^{\infty }}}U_{k}\left( t-t_{0}\right) ^{k}, \end{aligned}$$

where $U_{0}$, $U_{1}$, $U_{2}$, $\ldots$ are unknowns to be determined by DTM.

Applying the DTM to the initial condition (5) and equation ( 4) respectively, we obtain the transformed initial condition

7 $$\begin{aligned} U_{0}=u_{0}, \end{aligned}$$

and the recursion equation

8 $$\begin{aligned} kU_{k}=F\left( U_{0}, \ldots , U_{k-1},k-1\right) , \quad k=1,2,3,\ldots \end{aligned}$$

where $F\left( U_{0}, \ldots , U_{k-1},k-1\right)$ is the differential transforms of $f\left( u(t),t\right)$.

Using (7) and (8), we determine the unknowns $U_{k}$, $k=0,1,2$, $\ldots$ Then, the differential inverse transformation of the set of values $\left\{ U_{k}\right\} _{k=0}^{m}$ gives the approximate solution

9 $$\begin{aligned} u(t)=\displaystyle {{\sum _{k=0}^{m}}}U_{k}\left( t-t_{0}\right) ^{k}, \end{aligned}$$

where *m* is the approximation order of the solution. The exact solution of problem (4–5) is then given by (6).

If $U_{k}$ and $V_{k}$ are the differential transforms of *u*(*t*) and *v*(*t*) respectively, then the main operations of DTM are shown in Table 1.

**Table 1:** Main operations of DTM

Function	Differential transform
$\alpha u(t)\pm \beta v(t)$	$\alpha U_{k}\pm \beta V_{k}$
*u*(*t*)*v*(*t*)	${{\sum _{r=0}^{k}}}U_{r}V_{k-r}$
$\dfrac{d^{n}}{dt^{n}}[u(t) ]$	$k\left( k-1\right) \ldots \left( k+1-n\right) U_{k}$, $k\ge n$
$e^{\lambda t}$	$\dfrac{\lambda ^{k}e^{\lambda t_{0}}}{k!}$
$\sin \left( \omega t\right)$	$\dfrac{\omega ^{k}}{k!}\sin \left( \omega t_{0}+\dfrac{\pi k}{2}\right)$
$\cos \left( \omega t\right)$	$\dfrac{\omega ^{k}}{k!}\cos \left( \omega t_{0}+\dfrac{\pi k}{2}\right)$

The process of the DTM can be described as:

1. Apply the differential transform to initial condition (5).
2. Apply the differential transform to the differential equation ( 4) to obtain a recursion equation for the unknowns $U_{0}$, $U_{1}$, $U_{2}$, $\ldots$
3. Use the transformed initial condition (7) and the recursion equation (8) to determine the unknowns $U_{0}$, $U_{1}$, $U_{2}$, $\ldots$
4. Use the differential inverse transform formula (9) to obtain an approximate solution for initial-value problem (4– 5).

The solutions series obtained from DTM may have limited regions of convergence. Therefore, we propose to apply the Laplace–Padé resummation method to DTM truncated series to enlarge the convergence region as depicted in the next sections.

## Padé approximant

Given an analytical function *u*(*t*) with Maclaurin’s expansion

10 $$\begin{aligned} u\left( t\right) =\sum _{n=0}^{\infty }u_{n}t^{n}, 0\le t\le T. \end{aligned}$$

The Padé approximant to $u\left( t\right)$ of order [*L*, *M*] which we denote by $[L/M]_{u}\left( t\right)$ is defined by George A Baker et al. (1996)

11 $$\begin{aligned} \left[ L/M\right] _{u}\left( t\right) =\frac{p_{0}+p_{1}t+\ldots +p_{L}t^{L} }{1+q_{1}t+\ldots +q_{M}t^{M}}, \end{aligned}$$

where we considered $q_{0}=1$, and the numerator and denominator have no common factors.

The numerator and the denominator in (11) are constructed so that $u\left( t\right)$ and $[L/M]_{u}\left( t\right)$ and their derivatives agree at $t=0$ up to $L+M$. That is

12 $$\begin{aligned} u(t)-\left[ L/M\right] _{u}\left( t\right) =O\left( t^{L+M+1}\right) . \end{aligned}$$

From (12), we have

13 $$\begin{aligned} u\left( t\right) \sum _{n=0}^{M}q_{n}t^{n}-\sum _{n=0}^{L}p_{n}t^{n}=O\left( t^{L+M+1}\right) . \end{aligned}$$

From (13), we get the following algebraic linear systems

14 $$\begin{aligned} \left\{ \begin{array}{l} u_{L}q_{1}+\ldots +u_{L-M+1}q_{M}=-u_{L+1} \\ u_{L+1}q_{1}+\ldots +u_{L-M+2}q_{M}=-u_{L+2} \\ \vdots \\ u_{L+M-1}q_{1}+\ldots +u_{L}q_{M}=-u_{L+M}, \end{array} \right. \end{aligned}$$

and

15 $$\begin{aligned} \left\{ \begin{array}{l} p_{0}=u_{0} \\ p_{1}=u_{1}+u_{0}q_{1} \\ \vdots \\ p_{L}=u_{L}+u_{L-1}q_{1}+\ldots +u_{0}q_{L}. \end{array} \right. \end{aligned}$$

From (14), we calculate first all the coefficients $$q_{n},$$$$1\le n\le M$$. Then, we determine the coefficients $$p_{n},$$$$0\le n\le L$$ from (15).

Note that for a fixed value of $L+M+1$, the error (12) is smallest when the numerator and denominator of (11) have the same degree or when the numerator has degree one higher than the denominator.

## Laplace-Padé resummation method

Several approximate methods provide power series solutions (polynomial). Nevertheless, sometimes, this type of solutions lack large domains of convergence. Therefore, Laplace-Padé resummation method is used in literature to enlarge the domain of convergence of solutions or to find the exact solutions.

The Laplace-Padé method can be summarized as follows:

1. First, Laplace transformation is applied to power series (9).
2. Next, *s* is substituted by 1/*t* in the resulting equation.
3. After that, we convert the transformed series into a meromorphic function by forming its Padé approximant of order [*N*/*M*]. *N* and *M* are arbitrarily chosen, but they should be smaller than the order of the power series. In this step, the Padé approximant extends the domain of the truncated series solution to obtain better accuracy and convergence.
4. Then, *t* is substituted by 1/*s*.
5. Finally, by using the inverse Laplace *s* transformation, we obtain the exact or an approximate solution.

## Adomian polynomials and their relation with DTM

In this section, we briefly review the Adomian polynomials and their relation with the DTM. Usually a nonlinear term *N*(*u*) in a differential equation is decomposed in terms of Adomian polynomials $A_{n}$ (Rach 2008, 1984; Wazwaz 2000; Duan 2010a, b, 2011) as

16 $$\begin{aligned} N\left( u\right) =\sum _{n=0}^{\infty }A_{n}\left( u_{0},u_{1},\ldots ,u_{n}\right) , \end{aligned}$$

where $A_{n}$ are generated for all forms of nonlinearity from

17 $$\begin{aligned} A_{n}\left( u_{0},u_{1},\ldots ,u_{n}\right) =\frac{1}{n!}\frac{d^{n}}{ d\lambda ^{n}}\left[ N\left( \sum _{i=0}^{\infty }\lambda ^{i}u_{i}\right) \right] _{\lambda =0}, \quad n\ge 0, \end{aligned}$$

and where $$u_{n}\left( t\right) ,$$$$n=0,1,2,\ldots$$ denote the components used in the expansion

18 $$\begin{aligned} u\left( t\right) =\sum _{n=0}^{\infty }u_{n}\left( t\right) . \end{aligned}$$

There are several algorithms to compute Adomian polynomials but recently a convenient recursion to calculate Adomian polynomials for the *m*-variable case is proposed in (Duan 2011)

19 $$\begin{aligned} A_{n}=\dfrac{1}{n}\sum _{i=1}^{m}\sum _{k=0}^{n-1}\left( k+1\right) v_{i,k+1} \frac{\partial A_{n-1-k}}{\partial v_{i,0}},\quad n\ge 1. \end{aligned}$$

Also an extension of the differential transform to nonlinear terms of any type, known as the improved DTM, was given in (Fatoorehchi and Abolghasemi 2013a, 2014b) using Adomian polynomials

20 $$\begin{aligned} DT\{N(u)\}=A_{n}\left( U_{0},U_{1},\ldots ,U_{n}\right) , \end{aligned}$$

where $$U_{n}=DT\{u(t)\}.$$

In the coming sections, we make use of (19) and (20 ) to show how to solve nonlinear higher-index Hessenberg DAEs.

## Solution of higher-index Hessenberg DAEs by Adomian polynomials and DTM

In this section, we present our method for solving nonlinear higher-index Hessenberg differential-algebraic equations (DAEs). The technique is based on Adomian polynomials and the differential transform method (DTM). To solve the DAE, we first apply the DTM to it, where Adomian polynomials are used to compute the differential transforms of the nonlinear terms. The resulting recursion equations are rearranged in a nonsingular linear algebraic system for the coefficients of the power series solution. Two classes of nonlinear higher-index Hessenberg DAEs are solved.

### Higher-index nonlinear Hessenberg DAEs

The first class of higher-index Hessenberg DAEs we consider here is

21 $$\begin{aligned} u^{\left( m\right) }\left( t\right) = f\left( u\left( t\right) ,v\left( t\right) \right) , \end{aligned}$$

22 $$\begin{aligned} 0= g\left( u\left( t\right) \right) , \quad t\ge 0, \end{aligned}$$

where $$u^{\left( m\right) }\left( t\right)$$ denotes $$d^{m}u/dt^{m},$$*m*$$\ge 1$$ and $$u\in \mathbb {R}^{n_{u}}$$, $$v\in \mathbb {R}^{n_{v}}$$, $$g:\mathbb { R}^{n_{u}}\longrightarrow \mathbb {R}^{n_{v}},$$$$f:\mathbb {R}^{n_{u}}\times \mathbb {R}^{n_{v}}\longrightarrow \mathbb {R}^{n_{u}}$$.

The DAE is supplied with some consistent initial conditions

23 $$\begin{aligned} u^{\left( i\right) }\left( 0\right) =\eta _{i}, \quad i=0,\ldots ,m-1, \end{aligned}$$

$\eta _{i}$ are given constants.

System (21–22) has index $(m+1)$ if the product of the Jacobians

24 $$\begin{aligned} \left( \dfrac{\partial g}{\partial u}\right) \left( \dfrac{\partial f}{ \partial v}\right) \in \mathbb {R}^{n_{v}}\times \mathbb {R}^{n_{v}} \end{aligned}$$

is nonsingular for $t\ge 0.$

An important subclass of system (21–22) consists of those DAEs arising from the simulation of constrained mechanical multibody systems. Such DAEs have the form

25 $$\begin{aligned} \ddot{u}\left( t\right)= f(u\left( t\right) )+\left( \dfrac{\partial g}{ \partial u}\right) ^{^{{\textsf {T}}}}v\left( t\right) , \end{aligned}$$

26 $$\begin{aligned} 0= g\left( u\left( t\right) \right) , \quad t\ge 0, \end{aligned}$$

where $u\left( t\right)$ is the vector of generalized coordinates, $\ddot{u} \left( t\right)$ is the vector that contains the system accelerations, $\partial g/\partial u$ is the Jacobian of *g*, $v\left( t\right)$ is the Lagrange multipliers vector and $f(u\left( t\right) )$ is the generalized forces vector.

A standard assumption for these DAEs is the full rank condition

27 $$\begin{aligned} \text {rank}\left( \dfrac{\partial g}{\partial u}\right) =n_{v}, \end{aligned}$$

which means that the constraint equations are linearly independent. If condition (27) is satisfied then

28 $$\begin{aligned} \left( \dfrac{\partial g}{\partial u}\right) \left( \dfrac{\partial g}{ \partial u}\right) ^{^{{\textsf {T}}}}\in \mathbb {R}^{n_{v}}\times \mathbb {R}^{n_{v}} \end{aligned}$$

is nonsingular and DAE (25–26) is index-three.

Let $$f\left( u,v\right) =\left( f^{1}\left( u,v\right) ,f^{2}\left( u,v\right) ,\ldots ,f^{n_{u}}\left( u,v\right) \right) ^{^{{\textsf {T}} }}$$, then using (19), the Adomian polynomials $$F_{k}^{j},$$$$j=1,\ldots ,n_{u}$$, $$k=0,1,2,\ldots$$ for the $$\left( n_{u}+n_{v}\right)$$-variable function $$f^{j}\left( u,v\right)$$ are given by

29 $$\begin{aligned} F_{0}^{j}= f^{j}\left( U_{1,0},\ldots ,U_{n_{u},0},V_{1,0},\ldots ,V_{n_{v},0}\right) , \end{aligned}$$

30 $$\begin{aligned} F_{k}^{j}= \frac{1}{k}\sum _{i=1}^{n_{u}}\sum _{l=1}^{k}lU_{i,l}\frac{ \partial F_{k-l}^{j}}{\partial U_{i,0}}+\frac{1}{k}\sum _{i=1}^{n_{v}} \sum _{l=1}^{k}lV_{i,l}\frac{\partial F_{k-l}^{j}}{\partial V_{i,0}}, \quad k\ge 1, \end{aligned}$$

where $U_{i,l}$ and $V_{i,l}$ are the differential transforms of $u_{i}$ and $v_{i}.$

Equation (30) can be written as

31 $$\begin{aligned} F_{k}^{j}=\frac{1}{k}\sum _{i=1}^{n_{u}}\sum _{l=1}^{k}lU_{i,l}\frac{\partial F_{k-l}^{j}}{\partial U_{i,0}}+\frac{1}{k}\sum _{i=1}^{n_{v}} \sum _{l=1}^{k-1}lV_{i,l}\frac{\partial F_{k-l}^{j}}{\partial V_{i,0}}+ \sum _{i=1}^{n_{v}}V_{i,k}\frac{\partial F_{0}^{j}}{\partial V_{i,0}}, \quad k\ge 1. \end{aligned}$$

In vector form, we have

32 $$\begin{aligned} F_{0}= f\left( U_{0},V_{0}\right) , \end{aligned}$$

33 $$\begin{aligned} F_{k}= \frac{1}{k}\sum _{l=1}^{k-1}l\left( \begin{array}{cc} \dfrac{\partial F_{k-l}}{\partial U_{0}}&\dfrac{\partial F_{k-l}}{\partial V_{0}} \end{array} \right) \left( \begin{array}{c} U_{l} \\ V_{l} \end{array} \right) +\left( \frac{\partial F_{0}}{\partial U_{0}}\right) U_{k}+\left( \frac{\partial F_{0}}{\partial V_{0}}\right) V_{k}, \quad k\ge 1, \end{aligned}$$

where $$F_{k}=\left( F_{k}^{1},\ldots ,F_{k}^{n_{u}}\right) ^{^{{\textsf { T}}}},$$$$U_{k}=\left( U_{1,k},\ldots ,U_{n_{u},k}\right) ^{^{{\textsf {T} }}},$$$$V_{k}=\left( V_{1,k},\ldots ,V_{n_{v},k}\right) ^{^{{\textsf {T}} }},$$$$k=0,1,2\ldots$$

In a similar manner, let $$g\left( u\right) =\left( g^{1}\left( u\right) ,g^{2}\left( u\right) ,\ldots ,g^{n_{v}}\left( u\right) \right) ^{^{{ \textsf {T}}}}$$ then the Adomian polynomials $$G_{k}^{j},$$$$j=1,\ldots ,n_{v}$$ , $$k=0,1,2,\ldots$$ for the $$n_{u}$$-variable function $$g^{j}\left( u\right)$$ are given by

34 $$\begin{aligned} G_{0}^{j}= g^{j}\left( U_{1,0},\ldots ,U_{n_{u},0}\right) , \end{aligned}$$

35 $$\begin{aligned} G_{k}^{j}= \frac{1}{k}\sum _{i=1}^{n_{u}}\sum _{l=1}^{k-1}lU_{i,l}\frac{ \partial G_{k-l}^{j}}{\partial U_{i,0}}+\sum _{i=1}^{n_{u}}U_{i,k}\frac{ \partial G_{0}^{j}}{\partial U_{i,0}}, \quad k\ge 1. \end{aligned}$$

In vector form, we have

36 $$\begin{aligned} G_{0}= g\left( U_{0}\right) , \end{aligned}$$

37 $$\begin{aligned} G_{k}= \frac{1}{k}\sum _{l=1}^{k-1}l\left( \dfrac{\partial G_{k-l}}{ \partial U_{0}}\right) U_{l}+\left( \frac{\partial G_{0}}{\partial U_{0}} \right) U_{k}, \quad k\ge 1, \end{aligned}$$

where $$G_{k}=\left( G_{k}^{1},\ldots ,G_{k}^{n_{v}}\right)^{^{{\textsf { T}}}}.$$

To solve DAE (21–22), we apply the DTM to get

38 $$\begin{aligned} \left\{ \begin{array}{l} \alpha U_{k}=F_{k-m}, \\ 0=G_{k},\quad k\ge m, \end{array} \right. \end{aligned}$$

and

39 $$\begin{aligned} U_{k}=\eta _{k}, k=0,\ldots ,m-1, \end{aligned}$$

where $U_{k}$ is the differential transform of $u\left( t\right)$ and $\alpha =k\left( k-1\right) \ldots \left( k+1-m\right)$.

From (38), we obtain the linear algebraic recursion system

40 $$\begin{aligned} \left\{ \begin{array}{l} \alpha U_{k}-\left( \dfrac{\partial F_{0}}{\partial V_{0}}\right) V_{k-m}=R_{k-m}-F_{k-m}, \\ -\left( \dfrac{\partial G_{0}}{\partial U_{0}}\right) U_{k} =S_{k},\quad k\ge m, \end{array} \right. \end{aligned}$$

where

41 $$\begin{aligned} R_{k}=\frac{1}{k}\sum _{l=1}^{k-1}l\left( \begin{array}{cc} \dfrac{\partial F_{k-l}}{\partial U_{0}}&\dfrac{\partial F_{k-l}}{\partial V_{0}} \end{array} \right) \left( \begin{array}{c} U_{l} \\ V_{l} \end{array} \right) +\left( \frac{\partial F_{0}}{\partial U_{0}}\right) U_{k}, \end{aligned}$$

and

42 $$\begin{aligned} S_{k}=\frac{1}{k}\sum _{l=1}^{k-1}l\left( \dfrac{\partial G_{k-l}}{\partial U_{0}}\right) U_{l}. \end{aligned}$$

System (40) can be decomposed as

43 $$\begin{aligned} \left\{ \begin{array}{l} \left( \dfrac{\partial G_{0}}{\partial U_{0}}\right) \left( \dfrac{\partial F_{0}}{\partial V_{0}}\right) V_{k-m}=-\alpha S_{k}-\left( \dfrac{\partial G_{0}}{\partial U_{0}}\right) \left( R_{k-m}-F_{k-m}\right) , \\ \alpha U_{k}=\left( \dfrac{\partial F_{0}}{\partial V_{0}}\right) V_{k-m}+R_{k-m}-F_{k-m},\quad k\ge m. \end{array} \right. \end{aligned}$$

Since condition (24) holds, then the first equation of (43) can be solved uniquely for $V_{k-m}.$ Then using the second equation of (43), we can determine $U_{k}$. Therefore, an approximate analytical solution is given by

44 $$\begin{aligned} u(t)=\displaystyle {{\sum _{k=0}^{n}}}U_{k}t^{k}, \quad v(t)=\displaystyle {{ \sum _{k=0}^{n-m}}}V_{k}t^{k}. \end{aligned}$$

### Index-three nonlinear Hessenberg DAEs

The second class of higher-index nonlinear Hessenberg DAEs we consider here is

45 $$\begin{array}{l} \dot{u}= f\left( u,v\right) , \nonumber \\ \dot{v}= h\left( u,v,w\right) , \nonumber \\ 0= g\left( u\right) , \quad t\ge 0, \end{array}$$

where $$u\in \mathbb {R}^{n_{u}}$$, $$v\in \mathbb {R}^{n_{v}}$$, $$w\in \mathbb {R} ^{n_{w}}$$, $$g:\mathbb {R}^{n_{u}}\longrightarrow \mathbb {R}^{n_{w}},$$$$f: \mathbb {R}^{n_{u}}\times \mathbb {R}^{n_{v}}\longrightarrow \mathbb {R} ^{n_{u}},$$$$h:\mathbb {R}^{n_{u}}\times \mathbb {R}^{n_{v}}\times \mathbb {R} ^{n_{w}}\longrightarrow \mathbb {R}^{n_{v}}$$.

The DAE is supplied with some consistent initial conditions

46 $$\begin{aligned} u\left( 0\right) =\eta _{0}, \quad v\left( 0\right) =\eta _{1}. \end{aligned}$$

System (45) is index-three if the product of the Jacobians

47 $$\begin{aligned} \left( \dfrac{\partial g}{\partial u}\right) \left( \dfrac{\partial f}{ \partial v}\right) \left( \dfrac{\partial h}{\partial w}\right) \in \mathbb {R }^{n_{v}}\times \mathbb {R}^{n_{v}} \end{aligned}$$

is nonsingular for $t\ge 0.$

Let us assume that *f*,  *g* and *h* are sufficiently smooth and that the Jacobian $\partial g/\partial u$ has full row rank [i.e. rank $\left( \partial g/\partial u\right) =n_{w}$] for $t\ge 0.$

Let $$f\left( u,v\right) =\left( f^{1}\left( u,v\right) ,f^{2}\left( u,v\right) ,\ldots ,f^{n_{u}}\left( u,v\right) \right) ^{^{{\textsf {T}} }}$$ then the Adomian polynomials $$F_{k}^{j},$$$$j=1,\ldots ,n_{u}$$, $$k=0,1,2,\ldots$$ for the $$\left( n_{u}+n_{v}\right)$$-variable function $$f^{j}\left( u,v\right)$$ are given by

48 $$\begin{aligned} F_{0}^{j}= f^{j}\left( U_{1,0},\ldots ,U_{n_{u},0},V_{1,0},\ldots ,V_{n_{v},0}\right) , \end{aligned}$$

49 $$\begin{aligned} F_{k}^{j}= \frac{1}{k}\sum _{i=1}^{n_{u}}\sum _{l=1}^{k}lU_{i,l}\frac{ \partial F_{k-l}^{j}}{\partial U_{i,0}}+\frac{1}{k}\sum _{i=1}^{n_{v}} \sum _{l=1}^{k}lV_{i,l}\frac{\partial F_{k-l}^{j}}{\partial V_{i,0}}, \quad k\ge 1. \end{aligned}$$

Equation (49) can be written as

50 $$\begin{aligned} F_{k}^{j}=\frac{1}{k}\sum _{i=1}^{n_{u}}\sum _{l=1}^{k}lU_{i,l}\frac{\partial F_{k-l}^{j}}{\partial U_{i,0}}+\frac{1}{k}\sum _{i=1}^{n_{v}} \sum _{l=1}^{k-1}lV_{i,l}\frac{\partial F_{k-l}^{j}}{\partial V_{i,0}}+ \sum _{i=1}^{n_{v}}V_{i,k}\frac{\partial F_{0}^{j}}{\partial V_{i,0}}, \quad k\ge 1.\text { } \end{aligned}$$

In vector form, we have

51 $$\begin{aligned} F_{0}= f\left( U_{0},V_{0}\right) , \end{aligned}$$

52 $$\begin{aligned} F_{k}= \frac{1}{k}\sum _{l=1}^{k-1}l\left( \begin{array}{cc} \dfrac{\partial F_{k-l}}{\partial U_{0}}&\dfrac{\partial F_{k-l}}{\partial V_{0}} \end{array} \right) \left( \begin{array}{c} U_{l} \\ V_{l} \end{array} \right) +\left( \frac{\partial F_{0}}{\partial U_{0}}\right) U_{k}+\left( \frac{\partial F_{0}}{\partial V_{0}}\right) V_{k}, \quad k\ge 1, \end{aligned}$$

where $$F_{k}=\left( F_{k}^{1},\ldots ,F_{k}^{n_{u}}\right) ^{^{{\textsf { T}}}},$$$$U_{k}=\left( U_{1,k},\ldots ,U_{n_{u},k}\right) ^{^{{\textsf {T} }}},$$$$V_{k}=\left( V_{1,k},\ldots ,V_{n_{v},k}\right) ^{^{{\textsf {T}} }},$$$$k=0,1,2\ldots$$

In a similar manner, let Let $$h\left( u,v,w\right) =\left( h^{1}\left( u,v,w\right) ,h^{2}\left( u,v,w\right) ,\ldots ,h^{n_{v}}\left( u,v,w\right) \right) ^{^{{\textsf {T}}}}$$ then the Adomian polynomials $$H_{k}^{j},$$$$j=1,\ldots ,n_{v}$$, $$k=0,1,2,\ldots$$ for the $$\left( n_{u}+n_{v}+n_{w}\right)$$-variable function $$h^{j}\left( u,v,w\right)$$ are given by

53 $$\begin{aligned} H_{0}^{j}= h^{j}\left( U_{1,0},\ldots ,U_{n_{u},0},V_{1,0},\ldots ,V_{n_{v},0},W_{1,0},\ldots ,W_{n_{w},0}\right) , \end{aligned}$$

54 $$\begin{aligned} H_{k}^{j}= \frac{1}{k}\sum _{l=1}^{k}\left( \sum _{i=1}^{n_{u}}lU_{i,l}\frac{ \partial H_{k-l}^{j}}{\partial U_{i,0}}+\sum _{i=1}^{n_{v}}lV_{i,l}\frac{ \partial H_{k-l}^{j}}{\partial V_{i,0}}+\sum _{i=1}^{n_{w}}lW_{i,l}\frac{ \partial H_{k-l}^{j}}{\partial W_{i,0}}\right) , \quad k\ge 1. \end{aligned}$$

Equation (54) can be written as

55 $$\begin{aligned} H_{k}^{j}=\frac{1}{k}\sum _{l=1}^{k-1}\left( \sum _{i=1}^{n_{u}}lU_{i,l}\frac{ \partial H_{k-l}^{j}}{\partial U_{i,0}}+\sum _{i=1}^{n_{v}}lV_{i,l}\frac{ \partial H_{k-l}^{j}}{\partial V_{i,0}}+\sum _{i=1}^{n_{w}}lW_{i,l}\frac{ \partial H_{k-l}^{j}}{\partial W_{i,0}}\right) \end{aligned}$$

56 $$\begin{aligned} +\sum _{i=1}^{n_{u}}U_{i,k}\frac{\partial H_{0}^{j}}{\partial U_{i,0}} +\sum _{i=1}^{n_{v}}V_{i,k}\frac{\partial H_{0}^{j}}{\partial V_{i,0}} +\sum _{i=1}^{n_{w}}W_{i,k}\frac{\partial H_{0}^{j}}{\partial W_{i,0}}, \quad k\ge 1. \end{aligned}$$

In vector form, we have

57 $$\begin{aligned} H_{0}=h\left( U_{0},V_{0},W_{0}\right) , \end{aligned}$$

58 $$\begin{aligned} H_{k}=\frac{1}{k}\sum _{l=1}^{k-1}l\left( \begin{array}{ccc} \dfrac{\partial H_{k-l}}{\partial U_{0}}&\dfrac{\partial H_{k-l}}{\partial V_{0}}&\dfrac{\partial H_{k-l}}{\partial W_{0}} \end{array} \right) \left( \begin{array}{c} U_{l} \\ V_{l} \\ W_{l} \end{array} \right) \nonumber \\ +\left( \frac{\partial H_{0}}{\partial U_{0}}\right) U_{k}+\left( \frac{ \partial H_{0}}{\partial V_{0}}\right) V_{k}+\left( \frac{\partial H_{0}}{ \partial W_{0}}\right) W_{k},\quad k\ge 1, \end{aligned}$$

where $$H_{k}=\left( H_{k}^{1},\ldots ,H_{k}^{n_{v}}\right) ^{^{{\textsf { T}}}}.$$

In a similar manner, let $$g\left( u\right) =\left( g^{1}\left( u\right) ,g^{2}\left( u\right) ,\ldots ,g^{n_{v}}\left( u\right) \right) ^{^{{ \textsf {T}}}}$$ then the Adomian polynomials $$G_{k}^{j},$$$$j=1,\ldots ,n_{v}$$ , $$k=0,1,2,\ldots$$ for the $$n_{u}$$-variable function $$g^{j}\left( u\right)$$ are given by

59 $$\begin{aligned} G_{0}= g\left( U_{0}\right) , \end{aligned}$$

60 $$\begin{aligned} G_{k}= \frac{1}{k}\sum _{l=1}^{k-1}l\left( \dfrac{\partial G_{k-l}}{ \partial U_{0}}\right) U_{l}+\left( \frac{\partial G_{0}}{\partial U_{0}} \right) U_{k}, \quad k\ge 1, \end{aligned}$$

where $$G_{k}=\left( G_{k}^{1},\ldots ,G_{k}^{n_{v}}\right) ^{^{{\textsf { T}}}}.$$

To solve DAE (45–46), we apply the DTM to get

61 $$\begin{aligned} \left\{ \begin{array}{l} kU_{k}=F_{k-1}, \\ kV_{k}=H_{k-1}, \\ 0=G_{k},\quad k\ge 1, \end{array} \right. \end{aligned}$$

and

62 $$\begin{aligned} U_{0}=\eta _{0}, V_{0}=\eta _{1}, \end{aligned}$$

where $$U_{k},V_{k}$$ and $$W_{k}$$ are the differential transforms of $$u\left( t\right) ,$$$$v\left( t\right)$$ and $$w\left( t\right)$$ respectively.

From the (61), we finally come to the linear recursion system

63 $$\begin{aligned} \left\{ \begin{array}{l} kU_{k}-\left( \dfrac{\partial F_{0}}{\partial V_{0}}\right) V_{k-1} =R_{k-1}-F_{k-1}, \\ kV_{k}-\left( \dfrac{\partial H_{0}}{\partial W_{0}}\right) W_{k-1}=R_{k-1}^{\prime }-G_{k-1}, \\ -\left( \dfrac{\partial G_{0}}{\partial U_{0}}\right) U_{k}=S_{k},\quad k\ge 1, \end{array} \right. \end{aligned}$$

where

64 $$\begin{aligned} R_{k}^{\prime }= \frac{1}{k}\sum _{l=1}^{k-1}l\left( \begin{array}{ccc} \dfrac{\partial G_{k-l}}{\partial U_{0}}&\dfrac{\partial G_{k-l}}{\partial V_{0}}&\dfrac{\partial G_{k-l}}{\partial W_{0}} \end{array} \right) \left( \begin{array}{c} U_{l} \\ V_{l} \\ W_{l} \end{array} \right) +\left( \frac{\partial G_{0}}{\partial U_{0}}\right) U_{k}+\left( \frac{ \partial G_{0}}{\partial V_{0}}\right) V_{k}. \end{aligned}$$

System (63) can be decomposed as

65 $$\begin{aligned} \left\{ \begin{array}{l} \left( \dfrac{\partial G_{0}}{\partial U_{0}}\right) \left( \dfrac{\partial F_{0}}{\partial V_{0}}\right) \left( \dfrac{\partial H_{0}}{\partial W_{0}} \right) W_{k-2}=-\left( \dfrac{\partial G_{0}}{\partial U_{0}}\right) \left( \dfrac{\partial F_{0}}{\partial V_{0}}\right) \left( R_{k-2}^{\prime }-G_{k-2}\right) \\ +k\left( k-1\right) S_{k}-\left( k-1\right) \left( \dfrac{\partial G_{0}}{ \partial U_{0}}\right) \left( R_{k-1}-F_{k-1}\right) , \quad k\ge 2, \\ \left( k-1\right) V_{k-1}=\left( \dfrac{\partial H_{0}}{\partial W_{0}} \right) W_{k-2}+R_{k-2}^{\prime }-G_{k-2}, \quad k\ge 2, \\ kU_{k}=\left( \dfrac{\partial F_{0}}{\partial V_{0}}\right) V_{k-1} +R_{k-1}-F_{k-1}, \quad k\ge 1. \end{array} \right. \end{aligned}$$

Since condition (47) holds, then the first equation of (65) can solved uniquely for $W_{k-2}.$ Then $V_{k-1}$ is obtained from the second equation of (65). Last, the unknown $U_{k}$ is obtained from the third equation of (65). Then, an approximate analytical solution is given by

66 $$\begin{aligned} u(t)=\displaystyle {{\sum _{k=0}^{n}}}U_{k}t^{k}, \quad v(t)=\displaystyle {{ \sum _{k=0}^{n-1}}}V_{k}t^{k}, \quad w(t)=\displaystyle {{\sum _{k=0}^{n-2}}} W_{k}t^{k}. \end{aligned}$$

## Cases study

In this section, we will demonstrate the effectiveness of proposed technique through two nonlinear higher-index Hessenberg DAEs.

### Example 1

Consider the following nonlinear index-three Hessenberg DAE describing the constrained motion of a particle to a circular track

67 $$\begin{array}{l} \ddot{u}_{1}= 2u_{2}-2u_{2}^{3}-u_{1}v, \nonumber \\ \ddot{u}_{2}= 2u_{1}-2u_{1}^{3}-u_{2}v, \nonumber \\ 0= u_{1}^{2}+u_{2}^{2}-1, \quad t\ge 0. \end{array}$$

System (67) is supplied with the following (consistent) initial conditions

68 $$\begin{aligned} u_{1}\left( 0\right) =1, \quad \dot{u}_{1}\left( 0\right) =0, \quad u_{2}\left( 0\right) =0, \quad \dot{u}_{2}\left( 0\right) =1. \end{aligned}$$

Note that no initial condition $v\left( 0\right)$ is given to the variable $v\left( t\right)$ as $v\left( 0\right)$ is pre-determined by the DAE and initial conditions (68). System (67) is index-three since three time differentiations of the algebraic equation (third equation) of (67) will lead to an ordinary differential equation for $v\left( t\right)$. As a consequence, this DAE system is difficult to solve numerically due to numerical instabilities.

Therefore, to solve (67–68), we apply the DTM to (67) and get the recursion

69 $$\begin{aligned} k\left( k-1\right) U_{1,k}= 2U_{2,k-2}-2A_{k-2}^{2}-{{\sum _{l=0}^{k-2}}} U_{1,k-2-l}V_{l}, \nonumber \\ k\left( k-1\right) U_{2,k}= 2U_{1,k-2}-2A_{k-2}^{1}-{{\sum _{l=0}^{k-2}}} U_{2,k-2-l}V_{l}, \quad k\ge 2, \nonumber \\ 0= {{\sum _{l=0}^{k}}}U_{1,l}U_{1,k-l}+U_{2,l}U_{2,k-l}-\delta \left( k\right) , \quad k\ge 0, \end{aligned}$$

where the differential transform of the nonlinear terms $$u_{i}^{3}\left( t\right) ,$$$$i=1,2$$ are replaced by the Adomian polynomials

$$\begin{aligned} A_{k-2}^{i}=\sum _{m=0}^{k-2}\sum _{l=0}^{m}U_{i,k-2-m}U_{i,m-l}U_{i,l}, \quad i=1,2. \end{aligned}$$

Then applying the DTM to initial conditions (68), we get

70 $$\begin{aligned} U_{1,0}=1, \quad U_{1,1}=0, \quad U_{2,0}=0, \quad U_{2,1}=1. \end{aligned}$$

For $k=0$ and $k=1,$ the third equation of (69) gives

$$\begin{array}{l} U_{1,0}^{2}+U_{2,0}^{2}= 1, \\ U_{1,0}U_{1,1}+U_{2,0}U_{2,1}= 0, \end{array}$$

which are satisfied by the transformed initial conditions (70).

Therefore, system (69) reduces to the nonsingular algebraic system for the unknowns $U_{1,k},U_{2,k}$ and $V_{k-2}$

71 $$\begin{aligned} k\left( k-1\right) U_{1,k}+U_{1,0}V_{k-2}= 2U_{2,k-2}-2A_{k}^{2}-{{ \sum _{l=0}^{k-3}}}U_{1,k-2-l}V_{l}, \nonumber \\ k\left( k-1\right) U_{2,k}+U_{2,0}V_{k-2}= 2U_{1,k-2}-2A_{k}^{1}-{{ \sum _{l=0}^{k-3}}}U_{2,k-2-l}V_{l}, \nonumber \\ 2U_{1,0}U_{1,k}+2U_{2,0}U_{2,k}= -{{\sum _{l=1}^{k-1}}} U_{1,l}U_{1,k-l}+U_{2,l}U_{2,k-l}, \quad k\ge 2. \end{aligned}$$

Using (70) and solving (71), we obtain the following values

72 $$\begin{aligned} U_{1,2k}&=\frac{\left( -1\right) ^{k}}{\left( 2k\right) !}, \quad U_{1,2k+1}=0, \quad k=1,\ldots ,4, \nonumber \\ U_{2,2k+1}&=\frac{\left( -1\right) ^{k}}{\left( 2k+1\right) !} , \quad U_{2,2k}=0, \quad k=1,\ldots ,4, \nonumber \\ V_{0}&=1, \quad V_{1}=2, \quad V_{3}=-\frac{4}{3}, \quad V_{5}=\frac{4}{15}, \quad V_{7}=-\frac{8}{315},\quad V_{2k}=0 , \quad k=1,2,3. \end{aligned}$$

From these values, we construct the approximate solution

73 $$\begin{aligned} u_{1}\left( t\right) ={{\sum _{k=0}^{9}}}U_{1,k}t^{k}, \quad u_{2}\left( t\right) ={{\sum _{k=0}^{9}}}U_{2,k}t^{k}, \quad v\left( t\right) ={{ \sum _{k=0}^{7}}}V_{k}t^{k}. \end{aligned}$$

Applying Laplace transform to $$u_{1}\left( t\right) ,$$$$u_{2}\left( t\right)$$ and $$v\left( t\right) ,$$ we get

74 $$\begin{aligned} \mathcal {L}\left[ u_{1}\left( t\right) \right] =\sum _{k=1}^{5}\frac{\left( -1\right) ^{k-1}}{s^{2k-1}}, \quad \mathcal {L}\left[ u_{2}\left( t\right) \right] =\sum _{k=1}^{5}\frac{\left( -1\right) ^{k-1}}{s^{2k}} , \quad \mathcal {L}\left[ v\left( t\right) \right] =\dfrac{1}{s} +\sum _{k=1}^{4}\frac{\left( -1\right) ^{k-1}2^{2k-1}}{s^{2k}}. \end{aligned}$$

For simplicity we let $s=1/t$, then we have

75 $$\begin{aligned} \mathcal {L}\left[ u_{1}\left( t\right) \right] =\sum _{k=1}^{5}\left( -1\right) ^{k-1}t^{2k-1}, \quad \mathcal {L}\left[ u_{2}\left( t\right) \right] =\sum _{k=1}^{5}\left( -1\right) ^{k-1}t^{2k},\quad \mathcal {L} \left[ v\left( t\right) \right] =t+\sum _{k=1}^{4}\left( -1\right) ^{k-1}2^{2k-1}t^{2k}. \end{aligned}$$

All of the [*L* / *M*] *t*-Padé approximants of (75) with *L*$\ge 1$ and *M*$\ge 1$ and $L+M\le 4$ yield

76 $$\begin{aligned} \left[ \dfrac{L}{M}\right] _{u_{1}}=\frac{t}{1+t^{2}}, \quad \left[ \dfrac{L}{M}\right] _{u_{2}}=\frac{t^{2}}{1+t^{2}}, \quad \left[ \dfrac{ L}{M}\right] _{v}=\frac{4t^{3}+2t^{2}+t}{1+4t^{2}}. \end{aligned}$$

Now since $t=1/s$, we obtain from (76)

77 $$\begin{aligned} \left[ \dfrac{L}{M}\right] _{u_{1}}=\frac{s}{1+s^{2}}, \quad \left[ \dfrac{L}{M}\right] _{u_{2}}=\frac{1}{1+s^{2}}, \quad \left[ \dfrac{L}{M }\right] _{v}=\frac{s^{2}+2s+4}{4s+s^{3}}. \end{aligned}$$

Finally, applying the inverse Laplace transform to (77) we get

78 $$\begin{aligned} u_{1}\left( t\right) =\cos t, \quad u_{2}\left( t\right) =\sin t, \quad v\left( t\right) =1+\sin 2t, \end{aligned}$$

which is the exact solution of DAE initial-value problem (67–68).

### Example 2

Consider the following nonlinear index-three Hessenberg DAE

79 $$\begin{array}{l} \dot{u}_{1}= 2v_{1}, \nonumber \\ \dot{u}_{2}= 2v_{2}, \nonumber \\ \dot{v}_{1}= -2v_{1}+e^{u_{2}}+w+\varphi _{1}\left( t\right) ,\nonumber \\ \dot{v}_{2}= 2v_{2}+e^{u_{1}}+w+\varphi _{2}\left( t\right) , \nonumber \\ 0= u_{1}+u_{2}-\varphi _{3}\left( t\right) , \quad 0\le t<1, \end{array}$$

where

$$\begin{aligned} \varphi _{1}\left( t\right) =-\frac{2t^{4}+2t^{3}+1}{2\left( 1+t\right) ^{2}} , \quad \varphi _{2}\left( t\right) =\frac{-2t^{4}+2t^{3}-1}{2\left( 1-t\right) ^{2}}, \quad \varphi _{3}\left( t\right) =\ln \left( 1-t^{2}\right) . \end{aligned}$$

System (79) is supplied with the following (consistent) initial conditions

80 $$\begin{aligned} u_{1}\left( 0\right) =u_{2}\left( 0\right) =0,\quad v_{1}\left( 0\right) =-v_{2}\left( 0\right) =1/2. \end{aligned}$$

Note that no initial condition $w\left( 0\right)$ is given to the variable $w\left( t\right)$ as $w\left( 0\right)$ is pre-determined by the DAE and initial conditions (80). System (79) is index-three since three time differentiations of the algebraic equation (fifth equation) of (79) will lead to an ordinary differential equation for $w\left( t\right)$. As a consequence, this DAE system is difficult to solve numerically due to numerical instabilities.

To solve (79–80), we first expand $\varphi _{1}\left( t\right)$ and $\varphi _{2}\left( t\right)$ in Taylor series

81 $$\begin{array}{l} \varphi _{1}\left( t\right)= -\frac{1}{2}+t-\frac{3}{2}t^{2}+t^{3}-\frac{3 }{2}t^{4}+2t^{5}-\frac{5}{2}t^{6}+3t^{7}-\frac{7}{2}t^{8}, \nonumber \\ \varphi _{2}\left( t\right)= -\frac{1}{2}-t-\frac{3}{2}t^{2}-t^{3}-\frac{3 }{2}t^{4}-2t^{5}-\frac{5}{2}t^{6}-3t^{7}-\frac{7}{2}t^{8},\nonumber \\ \varphi _{3}\left( t\right)= -t^{2}-\frac{1}{2}t^{4}-\frac{1}{3}t^{6}- \frac{1}{4}t^{8}. \end{array}$$

Then, we apply the DTM to (79) and get the recursion

82 $$\begin{array}{l} kU_{1,k}= 2V_{1,k-1}, \nonumber \\ kU_{2,k}= 2V_{2,k-1}, \nonumber \\ kV_{1,k}= -2V_{1,k-1}+A_{k-1}^{2}+W_{k-1}+{\Phi }_{1,k-1},\nonumber \\ kV_{2,k}= 2V_{2,k-1}+A_{k-1}^{1}+W_{k-1}+{\Phi }_{2,k-1}, \quad k\ge 1, \nonumber \\ 0= U_{1,k}+U_{2,k}-{\Phi }_{3,k}, \quad k\ge 0, \end{array}$$

where $${\Phi }_{i,k}$$ is the differential transform of $$\varphi _{i}\left( t\right)$$, for $$i=1,2,3$$ and where the differential transform of the nonlinear terms $$e^{u_{i}},$$$$i=1,2$$ are replaced by the Adomian polynomials $$A_{k}^{i}$$

$$\begin{aligned} A_{0}^{i}= e^{U_{i,0}}, \\ A_{1}^{i}= U_{i,1}e^{U_{i,0}}, \\ A_{2}^{i}= U_{i,2}e^{U_{i,0}}+\frac{1}{2}U_{i,1}^{2}e^{U_{i,0}}, \\ A_{3}^{i}= U_{i,3}e^{U_{i,0}}+U_{i,1}U_{i,2}e^{U_{i,0}}+\frac{1}{6} U_{i,1}^{3}e^{U_{i,0}}, \\ A_{4}^{i}= U_{i,4}e^{U_{i,0}}+U_{i,1}U_{i,3}e^{U_{i,0}}+\frac{1}{2} U_{i,2}^{2}e^{U_{i,0}}+\frac{1}{2}U_{i,1}^{2}U_{i,2}e^{U_{i,0}}+\frac{1}{24} U_{i,1}^{4}e^{U_{i,0}}, \\ A_{5}^{i}= U_{i,5}e^{U_{i,0}}+\left( U_{i,2}U_{i,3}+U_{i,1}U_{i,4}\right) e^{U_{i,0}}+\frac{1}{2}\left( U_{i,1}U_{i,2}^{2}+U_{i,1}^{2}U_{i,3}\right) e^{U_{i,0}} \\&\quad+\frac{1}{6}U_{i,1}^{3}U_{i,2}e^{U_{i,0}}+\frac{1}{120} U_{i,1}^{5}e^{U_{i,0}}, \\ A_{6}^{i}= U_{i,6}e^{U_{i,0}}+\left( \frac{1}{2} U_{i,3}^{2}+U_{i,2}U_{i,4}+U_{i,1}U_{i,5}\right) e^{U_{i,0}}+\left( \frac{1}{ 6}U_{i,2}^{3}+U_{i,1}U_{i,2}U_{i,3}+\frac{1}{2}U_{i,1}^{2}U_{i,4}\right) e^{U_{i,0}} \\&\quad+\left( \frac{1}{4}U_{i,1}^{2}U_{i,2}^{2}+\frac{1}{6}U_{i,1}^{3}U_{i,3} \right) e^{U_{i,0}}+\frac{1}{24}U_{i,1}^{4}U_{i,2}e^{U_{i,0}}+\frac{1}{720} U_{i,1}^{6}e^{U_{i,0}}. \end{aligned}$$

Then, we apply the DTM to initial conditions (80), to get

83 $$\begin{aligned} U_{1,0}=U_{2,0}=0, \quad V_{1,0}=-V_{2,0}=1/2. \end{aligned}$$

Using the first two equations of (82) with $k=1$ and (83), we get

84 $$\begin{aligned} U_{1,1}=1, \quad U_{2,1}=-1. \end{aligned}$$

For $k=0$ and $k=1,$ the last equation of (82) gives

85 $$\begin{aligned} 0= U_{1,0}+U_{2,0}, \nonumber \\ 0= U_{1,1}+U_{2,1}, \end{aligned}$$

which are satisfied by (83) and (84).

Therefore, system (82) reduces to the following nonsingular linear algebraic system for the unknowns $U_{1,k},U_{2,k},V_{1,k-1},V_{2,k-1}$ and $W_{k-2}$

86 $$\begin{array}{l} V_{1,k-1}= \frac{1}{2}kU_{1,k}, \nonumber \\ V_{2,k-1}= \frac{1}{2}kU_{2,k}, \nonumber \\ \frac{1}{2}k\left( k-1\right) U_{1,k}-W_{k-2}= -2V_{1,k-2}+A_{k-2}^{2}+{ \Phi }_{1,k-2}, \nonumber \\ \frac{1}{2}k\left( k-1\right) U_{2,k}-W_{k-2}= 2V_{2,k-2}+A_{k-2}^{1}+{ \Phi }_{2,k-2}, \nonumber \\ 0= U_{1,k}+U_{2,k}+\frac{1+\left( -1\right) ^{k}}{k}, \quad k\ge 2. \end{array}$$

Adding the third and the fourth equations and using the last equation, we obtain $W_{k-2}.$ Now replacing $W_{k-2}$ by its expression in third and fourth equations, we get $U_{1,k}$ and $U_{2,k}.$ Last, we use the first and second equations to obtain $V_{1,k-1}$ and $V_{2,k-1}.$ Following this procedure and using (83) and (84), we obtain the approximations

87 $$\begin{aligned} u_{1}\left( t\right)&=t-\frac{1}{2}t^{2}+\frac{1}{3}t^{3}-\frac{1}{2}t^{4}+ \frac{1}{5}t^{5}-\frac{1}{6}t^{6}, \nonumber \\ u_{2}\left( t\right)&=-t-\frac{1}{2}t^{2}-\frac{1}{3}t^{3}-\frac{1}{2} t^{4}-\frac{1}{5}t^{5}-\frac{1}{6}t^{6}, \nonumber \\ v_{1}\left( t\right)&=\frac{1}{2}-\frac{1}{2}t+\frac{1}{2}t^{2}- \frac{1}{2}t^{3}+\frac{1}{2}t^{4}-\frac{1}{2}t^{5},\nonumber \\ v_{2}\left( t\right)&=-\frac{1}{2}-\frac{1}{2}t-\frac{1}{2} t^{2}-\frac{1}{2}t^{3}-\frac{1}{2}t^{4}-\frac{1}{2}t^{5}, \nonumber \\ w\left( t\right)&=t^{2}, \end{aligned}$$

which are the first terms of the Taylor series of the exact solutions

88 $$\begin{array}{l} u_{1}\left( t\right)= \ln (1+t), \quad u_{2}\left( t\right) =\ln (1-t) , \nonumber \\ v_{1}\left( t\right)= \frac{1}{2\left( 1+t\right) }, \quad v_{2}\left( t\right) =-\frac{1}{2\left( 1-t\right) }, \quad w\left( t\right) =t^{2}, \end{array}$$

of DAE initial-value problem (79–80).

## Discussion

Higher-index differential-algebraic equations (DAEs) still require new numerical and analytical methods to solve them efficiently. Such problems are known to be difficult to solve both numerically and analytically. In this paper, we introduced a new analytical method to solve nonlinear higher-index Hessenberg DAEs. The method is based on Adomian polynomials and the differential transform method (DTM). Two classes of nonlinear higher-index Hessenberg DAEs were treated by this method. The method has successfully handled these two classes of DAEs without the need for a preprocessing step of index-reduction. The method transformed the DAEs into easily solvable linear algebraic systems for the coefficient of the power series solution. For each class, one test problem was solved. The examples show that Adomian polynomials combined with the DTM are powerful tools to obtain the exact solutions or approximate solutions of nonlinear higher-index Hessenberg DAEs. To improve the power series solution, a Laplace-Padé post-treatement is applied to the truncated series leading to the exact solution.

## Conclusion

This work presents the analytical solution of two classes of nonlinear higher-index Hessenberg DAEs using Adomian polynomials and the DTM. Procedures for solving these two classes of DAEs are presented. For each class, the technique was tested on one nonlinear higher-index Hessenberg problem. The results obtained show that the method can be applied to solve nonlinear higher-index Hessenberg DAEs efficiently obtaining the exact solution or an approximate solution. On the one hand, it is important to note that these types of DAEs are difficult to solve both numerically and analytically. On the other hand, the presented technique based on Adomian polynomials and the DTM in combination with Laplace-Padé resummation method was able to obtain the exact solution of nonlinear higher-index Hessenberg DAEs. The use of Adomian polynomials allowed us to obtain an algorithm for the method and also to compute the differential transforms of highly nonlinear terms. The technique is based on a straightforward procedure that can be programmed in Maple or Mathematica to simulate large problems. Finally, future work is needed to apply the proposed technique to higher-index partial differential-algebraic equations and other nonlinear higher-index DAEs. Our method can be combined with the multi-stage DTM to calculate accurate approximate solutions to these problems.
